# Change of soil microbial community under long-term fertilization in a reclaimed sandy agricultural ecosystem

**DOI:** 10.7717/peerj.6497

**Published:** 2019-02-27

**Authors:** Zengru Wang, Yubing Liu, Lina Zhao, Wenli Zhang, Lichao Liu

**Affiliations:** 1 Shapotou Desert Research & Experiment Station, Northwest Institute of Eco-Environment and Resources, Chinese Academy of Sciences, Lanzhou, China; 2 Key Laboratory of Stress Physiology and Ecology in Cold and Arid Regions of Gansu Province, Northwest Institute of Eco-Environment and Resources, Chinese Academy of Sciences, Lanzhou, China

**Keywords:** Soil microbial communities, Long-term fertilization, Microbial abundance, Soil enzyme activity, Crop yield, Soil physicochemical properties

## Abstract

The importance of soil microbial flora in agro-ecosystems is well known, but there is limited understanding of the effects of long-term fertilization on soil microbial community succession in different farming management practices. Here, we report the responses of soil microbial community structure, abundance and activity to chemical (CF) and organic fertilization (OF) treatments in a sandy agricultural system of wheat-maize rotation over a 17-year period. Illumina MiSeq sequencing showed that the microbial community diversity and richness showed no significant changes in bacteria but decreased in fungi under both CF and OF treatments. The dominant species showing significant differences between fertilization regimes were Actinobacteria, Acidobacteria and Ascomycota at the phylum level, as well as some unclassified genera of other phyla at the genus level. As expected, soil organic matter content, nutrient element concentrations and bacterial abundance were enhanced by both types of fertilization, especially in OF, but fungal abundance was inhibited by OF. Redundancy analysis revealed that soil enzyme activities were closely related to both bacterial and fungal communities, and the soil nutrient, texture and pH value together determined the community structures. Bacterial abundance might be the primary driver of crop yield, and soil enzyme activities may reflect crop yield. Our results suggest a relatively permanent response of soil microbial communities to the long-term fertilization regimes in a reclaimed sandy agro-ecosystem from a mobile dune, and indicate that the appropriate dosage of chemical fertilizers is beneficial to sandy soil sustainability.

## Introduction

A growing global demand for agricultural crops is one of the main challenges in the 21st century. The pursuit of high productivity, long-term sustainability and optimal resource use efficiency without negative effects in the restricted land available for agricultural cultivation has led to the emergence of a variety of management practices (Xin & Li, 2018). Because of the low level of soil nutrients, the productivity of sandy land agro-ecological systems in arid areas is mainly dependent on intensive agricultural management. Generally, controlling watering and fertilization is a basic farming practice in which chemical or organic fertilizers with a certain amount of water are used primarily to improve soil nutrients and hence crop productivity (Mayer et al., 2015). However, determining how to evaluate the soil quality and sustainability in such systems is a major issue in farming management. Many variables have been studied in relation to long-term system sustainability, including available nutrients (Oehl et al., 2004), soil carbon (C) or nitrogen (N) stock development (Bosshard et al., 2009), and soil microorganism abundance and biodiversity (Rillig, 2004). Soil functioning and sustaining soil fertility is largely governed by the decomposition activity of the microflora (Anderson, 2003). In recent years, soil microbial community structure, diversity and activity have been used as indicators of overall soil health and productivity potential (Rasmussen et al., 1998; Böhme, Langer & Böhme, 2005; Kaschuk, Alberton & Hungria, 2010; Mbuthia et al., 2015) because the development of microbial metagenomic technology facilitates comprehensive analysis of culturable and unculturable microorganisms (Lentendu et al., 2014; Eo & Park, 2016). In addition, shifts in microbial community structure and the abundance of various plant-beneficial and detrimental soil microorganisms have been shown to influence the productivity and stability of the agroecosystem (Francioli et al., 2016).

Because microorganisms play a leading role in soil development and preservation (Schloter et al., 2018), there is a demand for quick and reproducible microbial-based indicators. Such indicators should ideally describe organisms with key functions in the system or with key regulatory/connecting roles (so-called keystone species). Therefore, in the search for suitable soil quality indicators it is important to consider parameters in which the biotic–abiotic interlinkages would find their expression (Vestergaard et al., 2017). However, in light of the huge functional redundancy in most soil microbiomes and the poor understanding of the relationship between microbial community structure and soil function (Pereira e Silva et al., 2012), finding specific keystone markers is not a trivial task and there has been a long debate on the best method for their selection (Anderson, 2003; Sharma et al., 2010; Schloter et al., 2018). Furthermore, there is currently no consensus regarding what would constitute a reasonable set of proxies that together constitute a good framework at the microbial community level for soil quality assessments (Schloter et al., 2018).

The effects of farming management practices on changes in the soil microbial community (i.e., bacteria and fungi) have been extensively studied in different soils, but the results have differed. For example, most studies of long-term chemical fertilization have revealed that a significant increase in soil microbial biomass (Geisseler & Scow, 2014) and long-term organic and chemical fertilization result in a clear shift in bacterial and fungal community composition (Lentendu et al., 2014; Hartmann et al., 2015; Francioli et al., 2016). However, some field studies based on short-term application of N amendments have revealed no significant changes or opposite results (Lazcano et al., 2013), and both positive and negative effects of chemical fertilizers on soil microbial activities have been reported (Guo et al., 2011; Nannipieri et al., 2012). Although higher microbial community diversity and biomass have been observed in most of these studies during short-term organic fertilization (Marschner, Crowley & Yang, 2004; Lentendu et al., 2014), these observations differ from those in long-term experiments (Eo & Park, 2016) and cannot be used to evaluate the effects of different farming systems on soil quality and sustainability (Rasmussen et al., 1998).

To provide a comprehensive understanding of whether sandy fields will be healthy and sustainable after application of integrated farming management, changes in soil microbial community structure and activity and their relationships with soil characteristics and crop yield were analyzed in a 17-year fertilization treatment experiment. Illumina MiSeq sequencing was used to analyze the prokaryotic 16S rDNA and fungal 18S rDNA in different fertilization regimes (chemical fertilization, organic fertilization and no fertilization), as well as in uncultivated mobile sand as a control. Soil physicochemical properties, bacterial and fungal abundance, and the activity of microbial intracellular oxidizing enzymes and extracellular hydrolases involved in nitrogen and phosphorus cycles in the soil were also determined. We hypothesized that (i) the composition, abundance and activity of the soil microbiome are driven by different fertilization regimes in the primary successional process; (ii) soil nutrient content is the main determinant for bacterial and fungal structure, whereas organic fertilizers (farmyard manure, FYM) are more helpful for the development of microbial community; and (iii) shifts in the structure and abundance of soil microorganisms affect the agro-ecosystem crop yield.

## Materials & Methods

### Study site, experimental design and soil sampling

The study site was in the southeast fringe of the Tengger Desert, at the Shaptou Desert Research and Experiment Station, Chinese Academy of Sciences (37°32′N, 105°02′E; elevation 1,340 m). This site is in a semiarid area with a mean annual precipitation of less than 200 mm. The local climate is that of a typical temperate continental desert and the area has a mean annual temperature of 9.6 °C and an average wind velocity of 3.5 m/s. The selected farm field was established in 2000 on mobile sand consisting of 1.0% silt, 20.01% clay and 78.99% sand from truncated sand dunes.

Three different treatments (no fertilization, C; chemical fertilization, CF; and organic fertilization, OF) were used in a fertilization experiment with wheat and maize rotation every year, and no planted sand was used as a control (CK). The size of each experimental field was 666.6 m^2^, and three replicates with areas of 10 × 10 m^2^ were randomly set in one field. The sources of chemical fertilizers were urea for N, single super-phosphate for phosphorus (P) and muriate of potash for potassium (K), and soils fertilized with CF received an annual dose of 150:45:60 kg ha^−1^ year^−1^ of N/P/K. The fertilizer application norm and recommended dosage were based on the data of the Second Soil Fertility Census in China (http://vdb3.soil.csdb.cn/extend/jsp/introduction; Shen, 1998). For CF, 30% of the N and the full dose of P and K were applied before sowing, and the remaining 70% of N was applied as top dressing coinciding with irrigation during the crop growing season. For OF, FYM (bean cake and chicken manure with bedding) with 300–350 g moisture kg^−1^ and 4.5–4.8 g N kg^−1^, 1.3–1.4 g P kg^−1^ and 1.6–1.8 g K kg^−1^ on a dry weight basis was applied. The FYM was thoroughly incorporated into the top 30 cm of the soil three weeks before each crop was sown. To ensure that the same amount of N, P and K was applied between CF and OF, the amount of applied FYM was converted according to the content of N in CF, and deficiencies of P and K in CF were calculated and supplemented as described above.

Soil samples were collected from different treatment fields in October 2017, three months after harvest of the wheat crop. To decrease spatial heterogeneity, we collected five soil cores (20 cm in depth and 5 cm in diameter) from four vertices and a diagonal point crossing of a square of 10 × 10 m^2^ individually using a sterile sampler. The five obtained cores consisted of a mixed sample. Triplicate composite samples for each treatment were collected and preserved in an ice box, then taken to the laboratory and immediately sieved (by 1 mm) to remove stones and plant roots. For microbial DNA extraction, samples were stored at −80 °C. For soil bulk density, samples were dried at 105 °C. To measure the soil other physiochemical properties and soil enzyme activity, samples were dried in natural air.

### Determination of soil biogeochemical properties and crop agronomic characteristics

Soil bulk density (SD) was estimated using a metallic cutting ring of known internal volume. Water holding capacity (WHC) was determined using the Wilcox method (Colman, 1947). Silt and clay particle content (Silt + Clay) was measured by the simple sieving method. Soil pH was determined with a pH detector using a 1:5 soil-to-water mixture, and electrical conductivity was measured using a portable conductivity meter. The soil total carbon (TC) content was determined using an elemental analyzer (Vario Macro Cube Elementar EL III, Hanau, Germany). Soil organic matter (OM) content was measured using the wet combustion method described by Walkley & Black (1934), soil available nitrogen (AN) was evaluated as described by Øien & Selmer-Olsen (1980), and the available phosphorus (AP) content was determined using a rapid perchloric acid digestion procedure (Galanos & Kapoulas, 1966). The available potassium (AK) was assessed with the sodium tetraphenyl boron method (Cox et al., 1999).

The soil enzymes urease (UE) and alkaline phosphatase (AKP), which are involved in N and P cycling, respectively, were analyzed using well-known assays described by Tabatabai & Bremner (1972) and Taylor et al. (2002), respectively. Two kinds of intracellular enzymes, soil dehydrogenase (DHA) and catalase (CAT), were determined using the method described by Casida, Klein & Santoro (1964) and by back-titrating residual H_2_O_2_ with KMnO_4_, respectively. One unit of enzyme activity was defined as the amount of enzyme required to free 1 µg/µmol of substrate per hour per gram of soil under the experimental conditions.

Plant biomass and yield (including above ground biomass, underground biomass, seed yield and thousand seeds weight) were analyzed by the weighing method. Stem length was measured using a ruler, and leaf area index was determined with a CI-202 Portable Laser Leaf Area Meter (CID Bio-Science, Inc., Camas, WA, USA). All soil biogeochemical properties and crop agronomic characteristics were carried out in triplicate.

### DNA extraction and Illumina MiSeq sequencing of soil microorganisms

An E.Z.N.A Soil DNA Kit (Omega Bio-Tek, Norcross, GA, USA) was used to extract the total DNA from 1 g of each of the soil samples described above according to the manufacturer’s instructions. The obtained DNA was then examined on 1.0% agarose gels, and the concentration was determined with a NanoDrop 2000c spectrophotometer (Thermo Fisher Scientific, Waltham, MA, USA), and the purified DNA was then used for PCR. The universal primers for the 16S rRNA gene for prokaryotic microbes and the 18S rRNA gene for fungi were used in the PCR. The primer information, as well as detailed procedures describing the amplicon and Illumina MiSeq sequencing, are described in Supplemental Information 1. The raw reads have been submitted to the NCBI Sequence Read Archive database under accession number SRP148524 (BioSample accession: SAMN09229018 –SAMN09229029) for bacteria and SRP148525 (BioSample accession: SAMN09229048 –SAMN09229059) for fungi. After the raw reads were demultiplexed and filtered using QIIME (version 1.17), as described in Supplemental Information, all sequences were clustered to operational taxonomic units (OTUs) at 97% sequence identity using UPARSE (version 7.1; http://drive5.com/uparse/).

### Quantification of soil bacteria and fungi by qPCR

qPCR was conducted to determine the absolute 16S and ITS rRNA gene abundance using the primer set 27F (5′-AGAGTTTGATCCTGGCTCAG-3′) and 338R (5′-TGCTGCCTCCCGTAGGAGT-3′) for bacteria and ITS1F (5′- CTTGGTCATTTAGAGGAAGTAA-3′) and ITS2R (5′-GCTGCGTTCTTCATCGATGC-3′) for fungi, to quantify the total microbial abundance. The detailed procedures of qPCR are described in Supplemental Information 1. qPCR analysis of each sample was replicated at least six times.

### Statistical analysis

Alpha biodiversity indices analysis was used to assess the richness and diversity of soil microbial diversity. Hierarchical clustering analysis and principal co-ordinate analysis (PCoA) were conducted to reflect the overall differences in community compositions. The major soil characteristics contributing to the microbial community structure were evaluated with redundancy analysis (RDA), which was also used to identify the attributes of microbial diversity and abundance in relation to the crop yield. We excluded OTUs with fewer than 5% occurrences in RDA analysis, and RDA ordinations were performed using CANOCO version 4.5 (Wang et al., 2012). Nonparametric tests (Kruskal-Wallis H test) were conducted to identify differences in the composition of microbial communities at the phylum and genus levels among multiple samples (Li et al., 2015). The LSD test and one-way analysis of variance (ANOVA) were conducted in SPSS Version 19.0 (IBM Corp., Armonk, NY, USA) to identify significant differences among fertilization treatments. Other data were analyzed and plotted in Origin 8.0 (Origin Lab Corporation, Northampton, MA, USA).

## Results

### Soil biogeochemical properties and crop agronomic characteristics

The soil biogeochemical properties and crop agronomic characteristics differed markedly among fertilization strategies. Both CF and OF clearly improved the soil texture and increased the levels of soil nutrients such as OM, AN and AP, and the effects of OF on OM content were significant (Table 1). However, AK decreased under both types of fertilization treatments, especially in CF soil. The soil enzyme activity showed significantly different responses to long-term fertilization regimes. Specifically, DHA was upregulated significantly by CF, whereas UE was stimulated by OF, and AKP and CAT showed no significant differences among fertilization treatments. Fertilization also stimulated an increase in plant biomass and yield, which were significantly higher in CF than OF (Table S1).

**Table 1:** Soil physicochemical properties and soil enzyme activities under different treatments.

	CK	C	CF	OF
OM (g/kg)	4.77 ± 0.56^c^	11.78 ± 1.42^b^	16.81 ± 1.46^a^	17.57 ± 1.11^a^
AN (mg/kg)	20.95 ± 1.50^c^	36.83 ± 1.19^b^	51.56 ± 4.98^a^	49.43 ± 3.60^a^
AP (mg/kg)	7.38 ± 1.04^c^	14.36 ± 2.93^b^	48.81 ± 5.13^a^	45.75 ± 3.10^a^
AK (mg/kg)	80.33 ± 3.18^bc^	120 ± 5.77^a^	73.33 ± 3.33^c^	103.33 ± 3.75^ab^
SD (g/cm^3^)	1.37 ± 0.09^a^	1.3 ± 0.03^a^	1.29 ± 0.07^a^	1.19 ± 0.04^b^
pH	8.59 ± 0.08^a^	8.27 ± 0.08^ab^	8.06 ± 0.05^c^	8.15 ± 0.05^bc^
EC (ds/m)	0.15 ± 0.01^b^	0.18 ± 0.01^b^	0.25 ± 0.02^a^	0.22 ± 0.01^a^
Silt+Clay (%)	21.01 ± 0.46^c^	27.07 ± 2.09^b^	31.27 ± 1.55^ab^	37.06 ± 1.41^a^
WHC (%)	21.6 ± 0.56^b^	24.99 ± 0.39^a^	25.78 ± 0.47^a^	26.83 ± 0.31^a^
DHA (µg/h/g)	0.64 ± 0.01^d^	0.99 ± 0.05^c^	4.47 ± 0.22^a^	2.22 ± 0.14^b^
CAT (µmol/h/g)	0.048 ± 0.01^c^	0.31 ± 0.017^b^	0.41 ± 0.014^a^	0.34 ± 0.011^b^
UE (µg/h/g)	0.58 ± 0.012^d^	10.57 ± 0.24^c^	20.76 ± 0.98^b^	37.06 ± 3.03^a^
AKP (µmol/h/g)	0.067 ± 0.01^c^	0.22 ± 0.012^b^	0.28 ± 0.007^ab^	0.31 ± 0.013^a^

The significant differences in each parameter (mean ± SE) among different treatments were determined by one-way ANOVA (LSD test). Different letters indicate significant difference at *P* < 0.05.

### Microbial community diversity and richness

**Figure 1:**
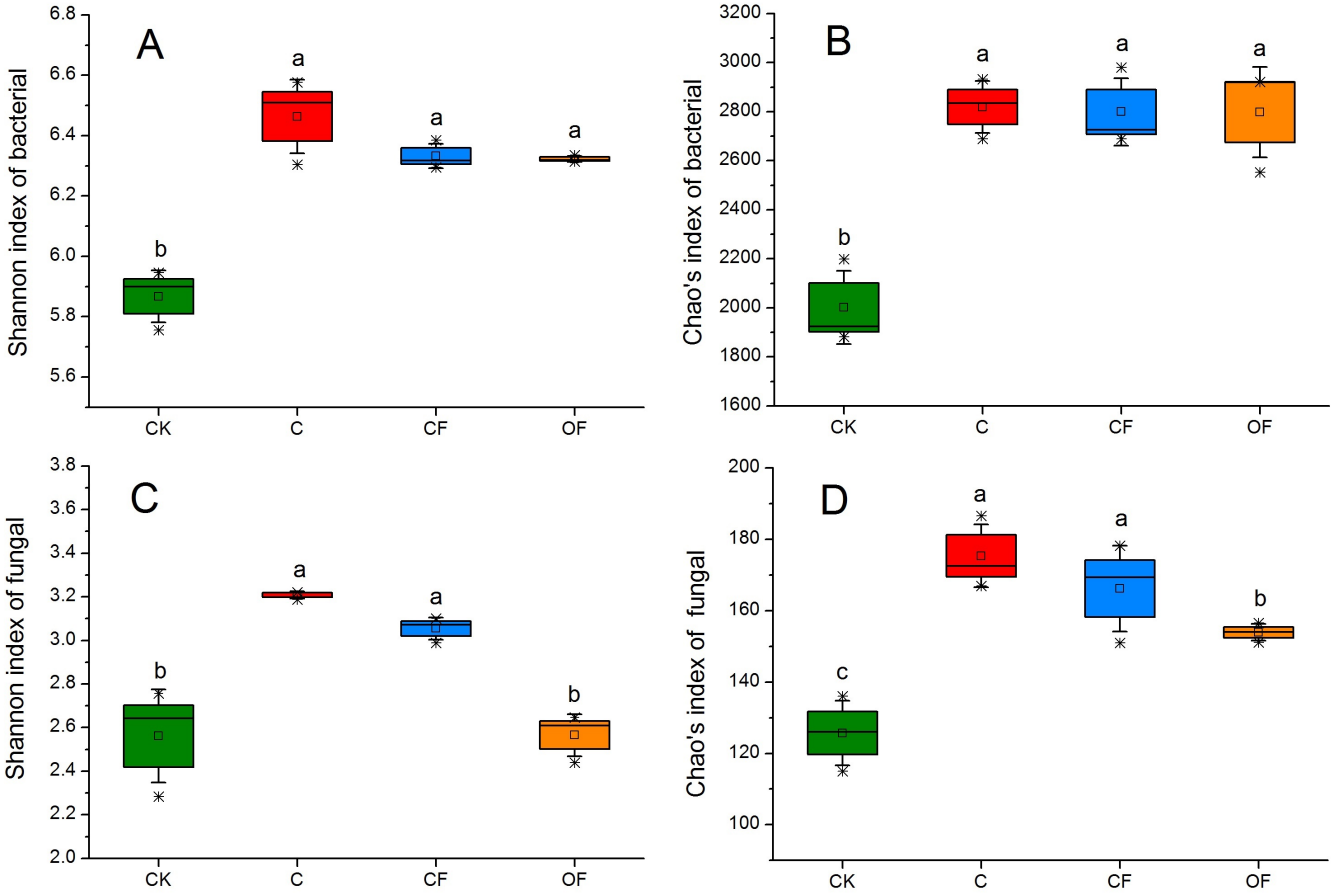
Box plots of the Shannon diversity (A, C) and Chao’s richness (B, D) indices at OTU level of bacterial and fungal community in the four soils studied. The differences among four soils were analyzed. Different letters indicate significant differences based on one-way ANOVA (LSD test) at *p* < 0.05.

**Figure 2:**
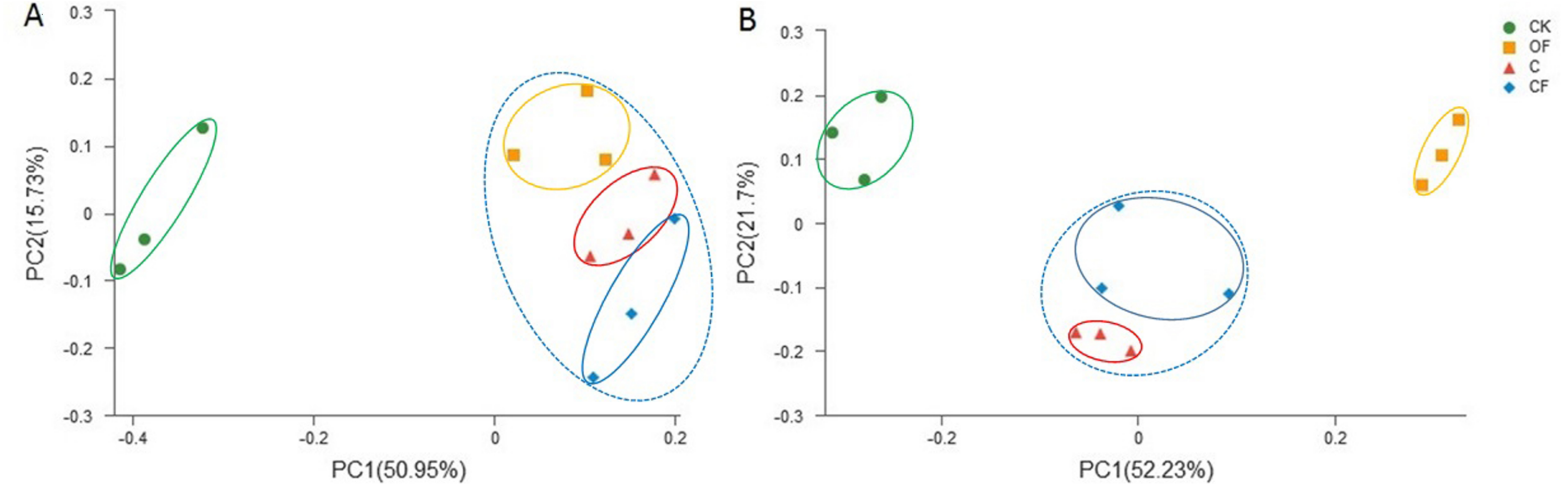
Principal coordinate analysis (PCoA) of (A) bacterial and (B) fungal community in the four soils studied at the OTU level based on 97% similarity.

To analyze the taxonomic compositions of the microbial community, a total of 627,173 16S rDNA and 437,477 18S fungal effective sequences with mean lengths of 273 bp and 401 bp, respectively, were identified by Illumina MiSeq sequencing in 12 soil samples (three replicates in four treatments). The sequences were clustered into 3,307 and 243 OTUs for prokaryotic microbes (Table S2) and fungi (Table S3) by using a threshold of 0.97, and there were at least 2,320 and 138 OTUs per sample in cultivated soils compared with 1,608 and 114 OTUs in CK, respectively. The most prokaryotic microbial OTUs were found in OF, whereas the most fungi were found in sample C, and 1,809 16S rDNA and 106 fungal OTUs were core species that comprised a relatively high proportion among all samples (60.20–84.53% and 54.40–70.67%, respectively) (Fig. S1), thus indicating a high community coherence among fertilization treatments. Estimation of community diversity and richness with the Shannon and Chao indexes, respectively, revealed that continuous fertilization for 17 years had no significant effects on the prokaryotic microbial community for any fertilization regime (Figs. 1A and 1B). However, the fungal richness and diversity decreased in the fertilization samples, especially in OF (Figs. 1C and 1D). Additionally, both the richness and diversity of the prokaryotic microbial and fungal community in farmed fields were higher than those in mobile sand. Furthermore, PCoA revealed that different fertilization regimes clustered into the same group on the basis of the prokaryotic community, as compared with CK (Fig. 2A). Groups were observed on the basis of fungal community and that of OF was significantly different from those of the other groups (Fig. 2B). Together, these findings indicated very different responses between prokaryotic microbial and fungal community structures after the application of different fertilization strategies.

### Bacterial and fungal abundance and dominant species

**Figure 3:**
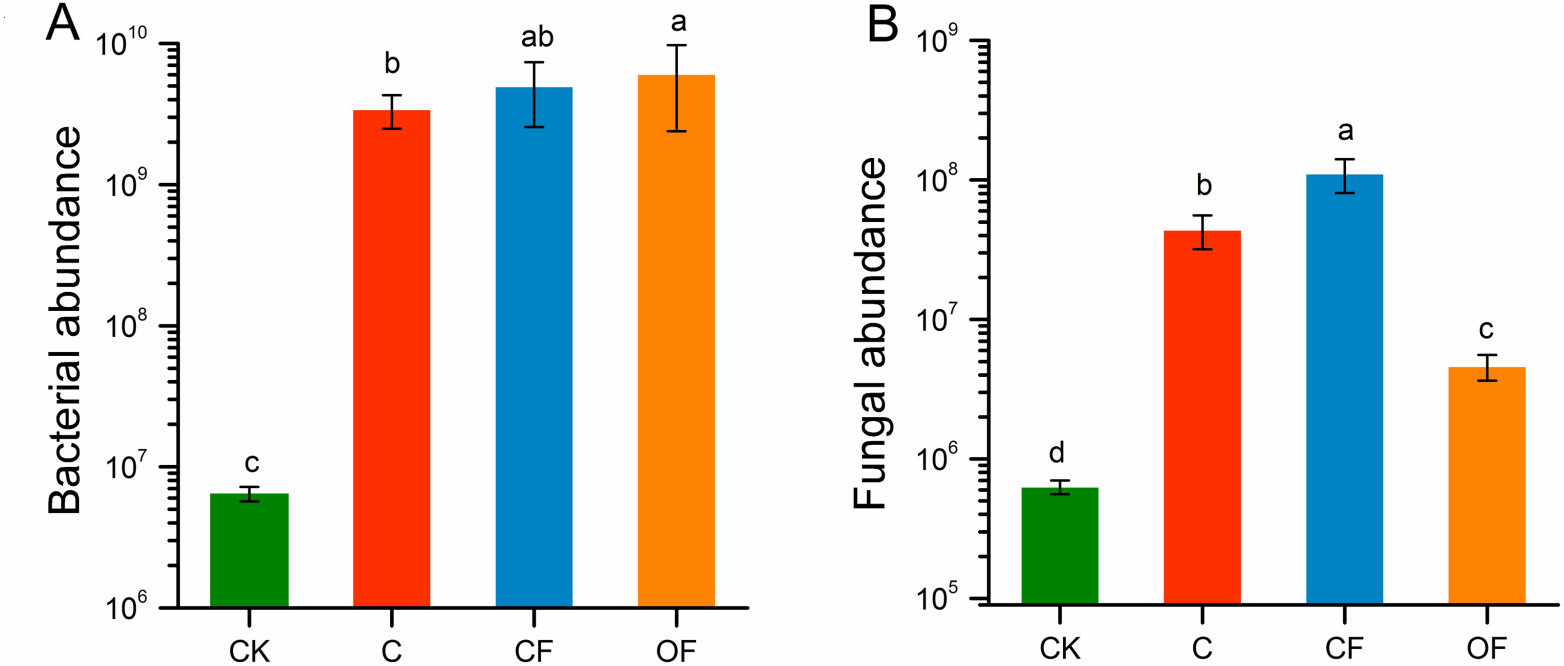
The absolute abundance of bacteria and fungi in the four soils studied. Different letters indicate significant differences based on LSD test at *p* < 0.05. Error bars represent one standard error of the mean (*n* = 6).

The qPCR indicated that the gene copy numbers of the bacterial 16S rRNA gene were significantly higher under fertilization conditions (both CF and OF) than no fertilization (*p* < 0.05), and were higher in OF than in CF (Fig. 3A). However, fungal ITS rRNA gene abundance was significantly stimulated by CF (*p* < 0.001) and decreased in OF relative to C (Fig. 3B). Both the bacterial and the fungal abundance in cultivated soils were higher than those in CK. These findings indicated that long-term fertilization promoted soil microbial community abundance and that applying organic fertilizer increased the total amount of microbes.

**Figure 4:**
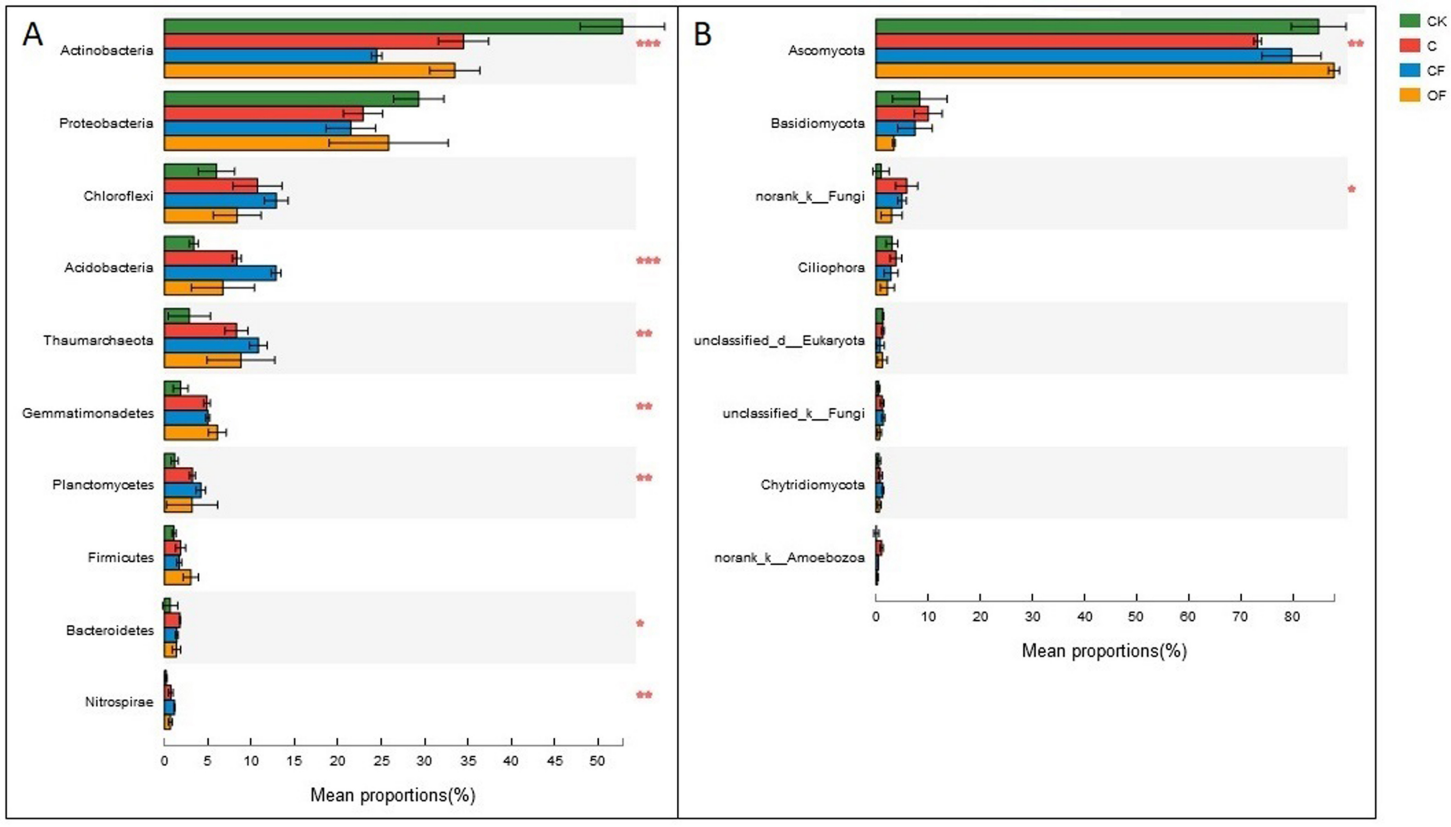
Dominant phyla (the relative abundance >1%) and the difference analysis of (A) bacteria and (B) fungi distributed in the four soils studied. Data are presented as mean ± standard deviation; *n* = 3 per soil sample. Kruskal–Wallis *H* test was used to assess the significance among samples. *P ≤ 0.05, **P ≤ 0.01, ***P < 0.001.

**Figure 5:**
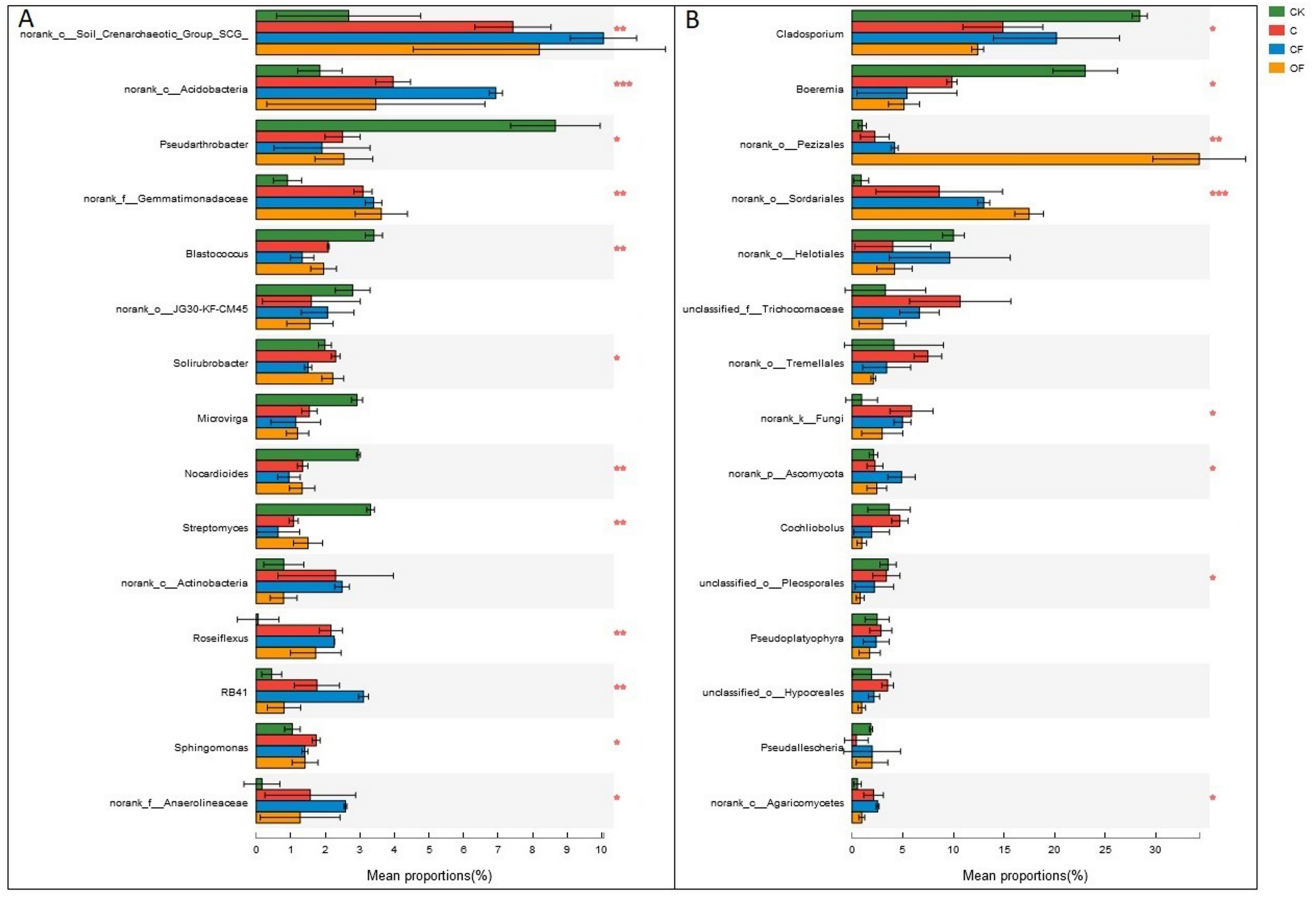
Dominant genera (the top 15) and the difference analysis of (A) bacteria and (B) fungi distributed in the four soils studied. Data are presented as mean ± standard deviation; *n* = 3 per soil sample. Kruskal–Wallis *H* test was used to assess the significance among soils. *P ≤ 0.05, **P ≤ 0.01, ***P < 0.001.

At the phylum level in the communities, a total of 30 prokaryotic microbial (Table S2) and 19 fungal phyla (Table S3) were retrieved at genetic distances of 3%, and the dominant phyla (relative abundance >10%) were Actinobacteria, Proteobacteria, Chloroflexi, Acidobacteria and Thaumarchaeota among prokaryotic microbes (Fig. 4A), and Ascomycota and Basidiomycota in fungi (Fig. 4B). No significant differences were found in Proteobacteria, Chloroflexi and Firmicutes among treatments (*p* < 0.05), and only two phyla (Ascomycota and an unclassified phylum) were significantly different in fungi. Notably, three unclassified genera of Crenarchaeota, Actinobacteria and Gemmatimonadaceae among prokaryotic microbes (Fig. 5A) and two unclassified genera of Pezizales and Sordariales in fungi (Fig. 5B) were the most abundant in the cultivated field. Twelve genera of prokaryotic microbes and eight genera of fungi among the 15 most abundant genera differed significantly among treatments, and the differences in fungi were mainly concentrated in highly abundant genera.

### Relationship between microbial community structure and soil properties

**Figure 6:**
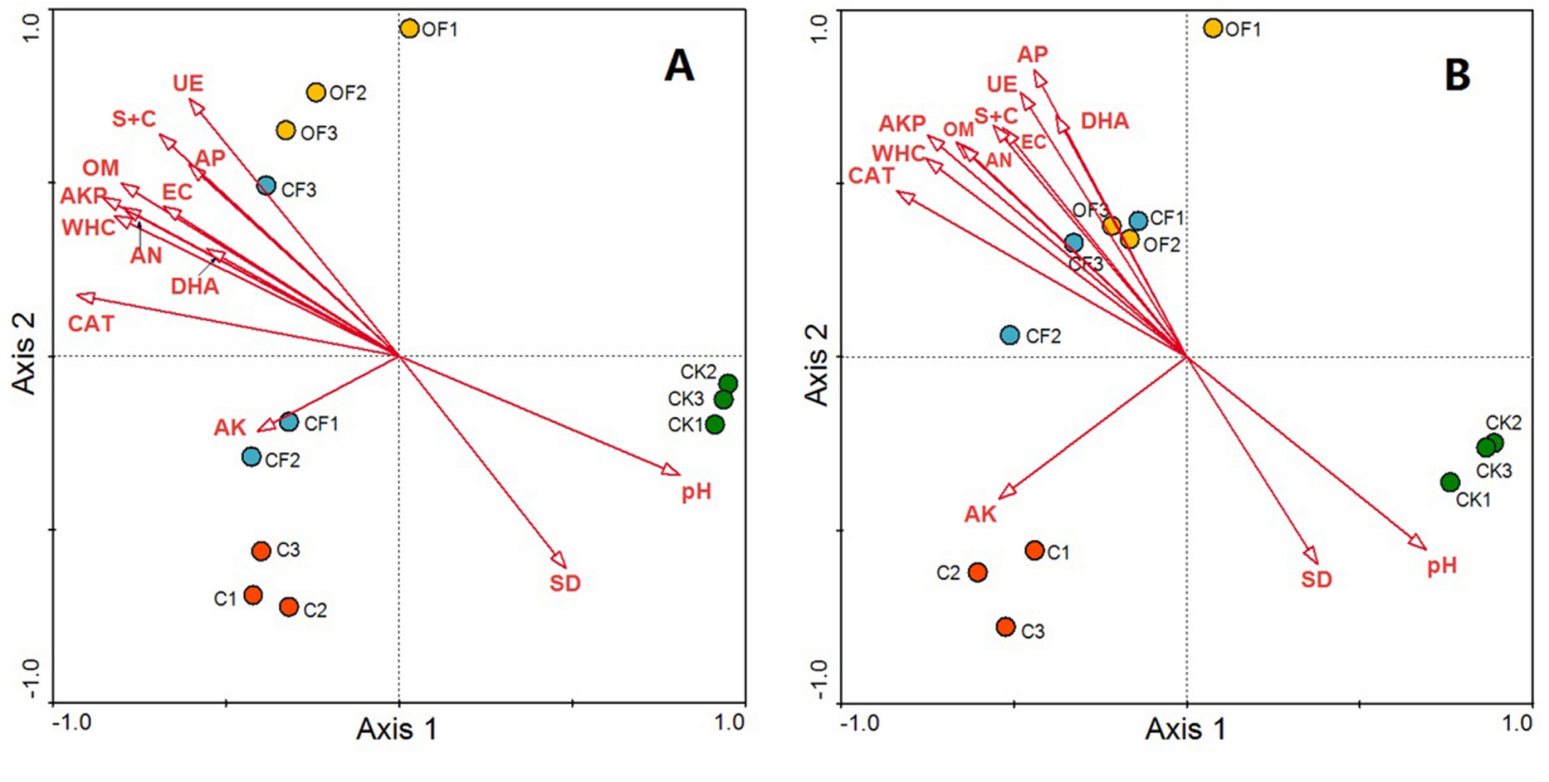
RDA ordination biplots of bacterial (A) and fungal (B) community in relation to soil environmental variables. Arrows indicate directions of maximum variation of environmental variables; length of arrows indicates their importance. Each circle represents the bacterial or fungal community for each sample. S + C, silt and clay content; SD, soil bulk density; WHC, water holding capacity; EC, electrical conductivity; OM, organic matter; AN, available nitrogen; AP, available phosphorus; AK, available potassium; DHA, dehydrogenase; CAT, catalase; AKP, alkaline phosphatase; UE, urease.

Redundancy analysis was used to identify distinct soil physicochemical properties and soil enzyme activities that explained changes in the prokaryotic microbial and fungal community structures. The total variation in prokaryotic and fungal community structures explained by the first four axes in the RDA (as constrained by the measured soil environmental variables) was 91.3% and 89.4%, respectively, with the first axis explaining 80.2% and 73.5% of the prokaryotic microbial and fungal changes, respectively, and the corresponding second axis explaining 7.6% and 11.9% (Fig. 6). According to a Monte Carlo permutation test, the first canonical axis was highly significant in both the prokaryotic microbial (F-ratio = 31.52; *p* < 0.01) and fungal community (F-ratio = 24.63; *p* < 0.01). Among the soil factors, soil pH, soil nutrient content (OM, AN and AP) and soil texture (silt and clay content, WHC) were strongly related to the prokaryotic microbial community structure, and soil enzyme activities (CAT, AKP, DHA and UE) also highly contributed to axis 1 (Fig. 6A). Thus, soil nutrient, texture and pH together determined prokaryotic microbial community structure, and shifts in community compositions directly influenced soil enzyme activities. The CF showed the highest scores along axis 1, whereas that of CK was the lowest. Additionally, OF and C were well separated along the second axis. These results indicated that the prokaryotic microbial community structure exhibited a clear gradient along the soil biogeochemical properties. The relationship between fungal community structure and soil properties was similar to that in the prokaryotic community, but soil nutrient content, soil texture and soil enzyme activity had a stronger influence on the fungal community structure (Fig. 6B).

### Relationship between crop agronomic characteristics and microbial community characteristics

**Figure 7:**
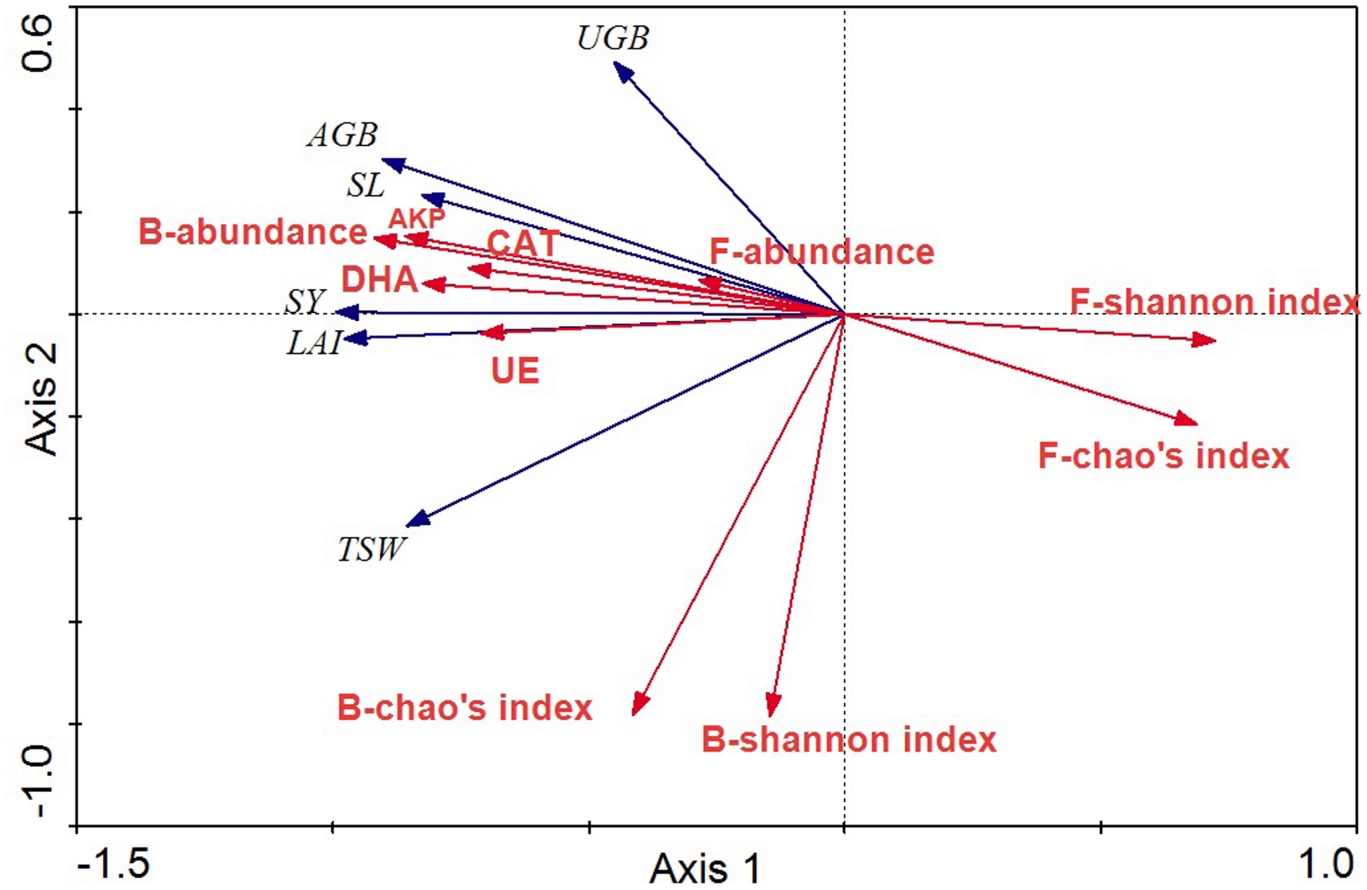
RDA ordination biplots of crop agronomic characteristics and microbial community characteristics. Arrows indicate directions of maximum variation of environmental variables. Length of arrows indicates their importance. Angles between arrows indicate correlations between variables. SY, seed yield; SL, stem length; LAI, leaf area index; AGB, above ground biomass; UGB, underground biomass; TSW, thousand seeds weight; DHA, dehydrogenase; CAT, catalase; AKP, alkaline phosphatase; UE, urease; B, bacteria; F, fungi.

The relationships between crop agronomic characteristics and microbial community characteristics (as environmental variables) were analyzed by RDA (Fig. 7). The first and second axes accounted for 91.0% and 3.0% of the total variations between crop agronomic characteristics and microbial community characteristics. There were significant effects of the microbial community and soil enzyme activities on crop yield in the Monte Carlo permutation test (F-ratio = 24.63; *p* < 0.01), and changes in crop agronomic characteristics were significantly correlated with bacterial abundance and fungal diversity (*p* < 0.05). The bacterial abundance was the major determinant for almost all crop yield indices except the underground biomass. The Shannon index of the fungal community was significantly and negatively correlated with above ground biomass and seed yield (*p* < 0.05), whereas soil enzyme activity was significantly and positively correlated with seed yield, stem length, leaf area index and above ground biomass.

## Discussion

Transformation from mobile sand to arable land in arid areas is good not only for economic returns but also for the future sustainable development of land in China. Therefore, reasonable management strategies without negative effects on soil quality and environmental systems are the main objectives of land development in agroecosystems. This study provides an overview of the responses of soil microbial communities to long-term fertilization from a traditional farming practice, and it could be useful in the development of methods of sustainable land management for newly developed land.

### Responses of the soil microbiome to long-term fertilization

Our first hypothesis that the composition, abundance and activity of the soil microbiome are driven by different fertilization regimes was verified by investigation of shifts in prokaryotic microbial and fungal community composition, abundance and soil enzyme activities between CF and OF. In the present study, long-term chemical and organic fertilization had significantly different effects on prokaryotic microbial and fungal community structures. There were no substantial changes in the prokaryotic microbial community diversity and richness; however, there were clear shifts in the proportion of prokaryotic microbial community compositions in relation to fertilization treatments. Fungal communities were much more sensitive to fertilization, showing a significant decrease in community diversity in response to organic fertilizers. These different responses in bacterial and fungal community structures have been reported in most previous studies of long-term fertilization treatments (Lentendu et al., 2014; Hartmann et al., 2015; Eo & Park, 2016) and even in a 113-year fertilization experiment (Francioli et al., 2016). Contrary to our expectations, the fungal community diversity was not enhanced by long-term organic fertilization. From the perspective of soil microbial community succession, PCoA ordination plots revealed significant effects on the microbial community structure after cultivation, and different fertilization management methods greatly affected the fungal community structure (Fig. 2).

Further study of the different proportions of dominant prokaryotic microbial and fungal groups responding to fertilization treatments at the phylum and genus levels (Figs. 4 and 5) revealed significant differences in all dominant phyla except Proteobacteria, Chloroflexi and Firmicutes in the prokaryotic communities between treatments, but Ascomycota were the only fungi affected by chemical and organic fertilization. Proteobacteria and Firmicutes, which are generally seen as copiotrophic bacteria (Lienhard et al., 2014) that prefer carbon-rich environments, are more abundant in FYM treatments than in systems subjected to long-term chemical fertilization (Francioli et al., 2016). However, our results showed that the OM and TN content were comparable between OF and CF, thus potentially leading to comparable growth proportions of these two phyla. Chloroflexi, which mainly consist of the filamentous anoxygenic phototrophic bacteria, is a thermophilic phylum that is well adapted to drought conditions (Hugenholtz and Stackebrandt, 2004) and is insensitive to fertilization treatments. Among bacterial phyla that differed slightly (*p* < 0.05) between treatments, Acidobacteria and Actinobacteria showed the most significant changes (*p* < 0.001). Actinobacteria, the predominant phylum in all soils investigated in this study, play a major role in agricultural soil quality promotion via organic matter decomposition (Strap, 2011) and these organisms were more abundant in the mobile sand and unfertilized soil. This phylum may consist of oligotrophs and may have a positive correlation with pH. In contrast, Acidobacteria were significantly lower in OF, a result that may be explained by the many reports of a negative correlation between this phylum and pH (Rousk et al., 2010; Fierer et al., 2012). Ascomycota, the predominant fungal phylum in agro-ecosystems and sandy soil (Lienhard et al., 2014), are important decomposers of organic substrates such as wood, leaf litter and dung, and thus showed the greatest diversity when applied to the FYM.

At the genus level, the universal primers of 16S rDNA revealed that the genera present in the highest proportion was the soil crenarchaeotic group, which consists of ammonia-oxidizing archaea and plays a major role in C fixation and nitrification of N metabolism (Zhang et al., 2012). Two unclassified genera in Actinobacteria and Gemmatimonadaceae were the most abundant bacteria after cultivation (Fig. 5A), whereas two unclassified genera in Pezizales and Sordariales of fungi accumulated in OF (Fig. 5B). These observations demonstrated that unknown specific functions should be considered in these species that differed significantly between treatments. In this light, knowledge of how fertilization strategies enhance the proliferation of taxa from high throughput sequencing is invaluable. Consequently, wet lab experiments are needed to increase understanding of the effects of specific management strategies on soil microorganisms and their interaction processes, to increase crop yields under sustainable agricultural systems.

Moreover, there were clear responses of microbial abundance and soil enzyme activities to long-term fertilization. The bacterial abundance was highest in OF, which is consistent with the soil organic matter and available potassium levels and indicates that the bacterial growth rate was closely related to soil fertility. Soil enzyme activity, a potential indicator of organic matter decomposition, is often affected by specific management strategies and plays important roles in sustainable agroecosystems. Soil dehydrogenase activity occurs intracellularly in all living microbial cells and is therefore commonly used as an indicator of overall soil microbial activity (Kaczynski et al., 2016). Catalase activity has been used as an indicator of soil fertility, because it is related to the metabolic activity of aerobic organisms (Trasar-Cepeda, Gil-Sotres & Leiros, 2007). The activity of both of these enzymes was highest in CF, indicating that CF promoted soil microbial activity and metabolic potential. Extracellular enzyme activities can be used as indicators of microbial nutrient demand because they are the proximate agents of organic matter decomposition (Sinsabaugh et al., 2010). UE and AKP, which are involved in N acquisition and P mineralization, respectively, had the highest activity in OF. This result can be interpreted as indicating that all extracellular enzyme activities are significantly related to soil pH and nutrient content, and that hydrolytic activities are more variable than oxidative activities in response to soil nutrients (Sinsabaugh et al., 2010).

### Correlation between soil microbiome and soil and crop characteristics

Our second hypothesis that soil nutrient content is the main determinant of microbial structure, and FYM is more beneficial for development of the microbial community, was partly verified by our analysis of prokaryotic microbial and fungal community structures according to soil pH, soil nutrients and texture. No significant differences were detected in the prokaryotic community structure between CF and OF, whereas FYM inhibited the fungal community diversity and richness.

Soil pH is an important factor that drives prokaryotic microbial community composition (Lauber et al., 2009). The application of mineral fertilizer significantly decreases the soil pH (Luo et al., 2015). Our analysis confirmed this result after 17 years of continuous application of mineral fertilizers and indicated that this effect may have been due to the release of hydrogen (H^+^) ions by oxidation of ammonium (${\mathrm{NH}}_{4}^{+}$), which is commonly used as a nitrogenous fertilizer in paddy cultivation (Kumar et al., 2017). However, the pH after FYM application was higher than that after chemical fertilization, probably because of enrichment of cations (Murugan & Kumar, 2013). Variations in pH between CF and OF affected the species that were closely related to soil pH, such as Acidobacteria and Actinobacteria. Application of FYM promoted a higher level of soil nutrient and texture with high OM, silt, clay and WHC than that in CF, possibly because of the amount of organic carbon and microbial biomass carbon in FYM added to the soil (Joergensen, Mäder & Fließbach, 2010). These changes in turn promoted the rapid propagation of bacteria and inhibited fungal community abundance and diversity in OF.

In recent years, some studies have shown that soil nutrient elements such as OC and TN are more closely correlated to the bacterial community structure than pH, whereas no correlations were observed between the fungal community and these elements (Zhong, Yan & Shangguan, 2015). Extensive studies of the effects of different agricultural management practices on the bacterial community have revealed that these communities are greatly influenced by soil physicochemical properties, whereas fungal communities have been shown to be unchanged or negatively affected by these practices (Luo et al., 2015). Moreover, short-term application of chemical and organic fertilizers have revealed no significant changes in microbial community (Lazcano et al., 2013), and most studies of long-term fertilization have revealed a clear shift in bacterial and fungal community composition (Lentendu et al., 2014; Francioli et al., 2016). Both positive and negative effects of organic and chemical fertilizers on soil microbial activity have been reported (Guo et al., 2011; Nannipieri et al., 2012) because of differences among crop plants and fertilization treatment times.

The third hypothesis, in which shifts in the structure and abundance of soil microorganisms affect the crop yield of the agro-ecosystem, was verified by our observation that bacterial abundance and fungal diversity indexes were closely related to crop yield. Many studies have attempted to link crop productivity to below-ground microorganisms and have addressed the potentially important role of bacterial taxa in soil OM accumulation, increases in soil enzyme activity, plant growth promotion and disease suppression in different fertilization regimes (Marschner, Crowley & Yang, 2004); however, other reports have shown reduced crop yield and biomass after continuous application of N and FYM (Kumar et al., 2017). Because of the limited knowledge of bacterial ecological function (Hartmann et al., 2015), it is difficult to use metagenomics to determine which beneficial or pathogenic taxa are promoted or suppressed in crop plants as a result of different fertilization regimes. In the present study, RDA analysis showed that bacterial abundance may be the most important positive factor influencing crop yield, and fungal diversity was negatively correlated to crop yield, indicating that specific types of fertilization can promote beneficial rather than detrimental groups of beneficial soil microorganisms. However, soil enzyme activities were closely correlated to prokaryotic microbial and fungal community structures in our study. Generally, soil enzyme activity may be a useful indicator of soil functional diversity, and it has been reported to provide a unique integrative biochemical assessment of soil function and condition (Chaparro et al., 2012). In this light, soil enzyme activity is very important for evaluating the crop productivity and sustainability of agricultural systems.

### Evaluation of soil sustainability

The living soil system, which provides several ecosystem services including nutrient cycling, water regulation and controlling pests and diseases (Sharma et al., 2010; Kumar et al., 2017), is of primary importance in sustainable agricultural production. Microorganisms are one of the originators of soil and play an important role in determining soil functions including decomposition of above- and below-ground plant material (Biswas & Kole, 2017). In addition, microbes provide an integrated measure of soil quality that cannot always be obtained from physical and chemical measures and/or analysis of higher organisms. Shifts in the structure and composition of the distinct microbial community are strong indicators of soil biological activity and crop productivity of terrestrial agro-ecosystems (Sharma et al., 2010); consequently, microbial analyses can discriminate soil quality status and utilization of shifts in microbial populations and activity and can be used as indicators of changes in soil quality. The use of microbial community structure and diversity as an indicator to monitor soil quality is challenging, because understanding of the relationship between community structure and soil function is lacking. Although the relationship between soil quality and microbial diversity is not completely understood, a moderate improvement in the soil environment accompanied by increasing microbial biomass and diversity in agricultural soil is generally considered to indicate ‘good’ soil quality. Our study demonstrated that soil microbial communities and crop agronomic traits were significantly altered by long-term fertilization, and that these shifts promoted the quality of agricultural systems.

Many studies have shown that organic fertilization stimulates the release of plant available nutrients in soil and enhances soil biological activities, thereby establishing a sustainable agricultural ecosystem (Carpenter-Boggs, Kennedy & Reganold, 2000). However, farming practices sometimes adversely affect soil biological properties, resulting in low soil quality (Böhme, Langer & Böhme, 2005). For example, fungal community structure and abundance play important roles in soil stability through a spatial hyphal network, which develops throughout the soil and can be strongly affected by fertilization and other agricultural management practices (Frey, Elliott & Paustian, 1999). As a result, the ecological functions of arbuscular mycorrhizal fungi as they relate to soil quality have garnered increased scientific attention in recent years (Rillig, 2004). In the present study, the soil microbial properties in CF showed a pattern of similar bacterial diversity and abundance, better fungal diversity and abundance, and higher microbial activities to that of OF; accordingly, the crop yield and biomass were also higher in CF. Thus, long-term sustainable soil management can be achieved in a fertilization system with chemical fertilizers, not just organic manure. The present experiment clearly shows the practicality of converting arid mobile sand to an arable system with a reasonable chemical fertilization level. The environmental and agronomic long-term optimum might lie between chemical and organic fertilization in an irrigable arid sandy system. Our data provide a good basis for avoiding the blind reduction of chemical fertilizers by considering the long-term sustainable development of arable land.

## Conclusions

Succession of microbial community characteristics and soil biogeochemical properties from an arid sandy system showed that both long-term chemical and organic fertilization enhanced the soil nutrient content available to plants, changed the microbial community structure, promoted soil enzyme activity, and increased crop plant yield. These results were compatible with our first hypothesis. However, contrary to our second hypothesis, no significant differences were observed in prokaryotic microbial community diversity in relation to fertilization regimes, and FYM inhibited the development of the fungal community. In contrast, chemical fertilization was more beneficial to bacterial propagation and crop yield, and bacterial abundance might be the main determinant of crop yield. Thus, changes in soil microbial activities and crop yield under long-term fertilization practices may be achieved mainly by bacteria rather than fungi. This field experiment indicates a relatively permanent response of soil bacterial and fungal communities in a sandy agro-ecosystem to long-term fertilization, and provides a comprehensive understanding of the effects of fertilization regimes on sustainable soil development. Overall, the results obtained from a sandy agricultural system managed by chemical and organic fertilization over a 17-year period indicated that it is possible to perform reasonable chemical fertilization management while maintaining soil health during farming practices.

##  Supplemental Information

10.7717/peerj.6497/supp-1 Supplemental Information 1 Supplementary methods

10.7717/peerj.6497/supp-2 Figure S1 Number of distinct and shared OTUs between bacterial (A) and fungal (B) community

10.7717/peerj.6497/supp-3 Table S1 Crop agronomic characteristics Details of crop agronomic characteristics under different fertilization treatments.

10.7717/peerj.6497/supp-4 Table S2 Bacterial OTU The OTU numbers of bacteria in different samples by using a threshold of 0.97.

10.7717/peerj.6497/supp-5 Table S3 Fungal OTUs The OTU numbers of bacteria in different samples by using a threshold of 0.97.

## Additional Information and Declarations

### Data Availability

The following information was supplied regarding data availability: The raw reads are available at the NCBI Sequence Read Archive database: accession number SRP148524 (BioSample accession: SAMN09229018 –SAMN09229029) for bacteria and SRP148525 (BioSample accession: SAMN09229048 –SAMN09229059) for fungi. After the raw reads were demultiplexed and filtered using QIIME (version 1.17), as described in Supplemental Information, all sequences were clustered to operational taxonomic units (OTUs) at 97% sequence identity using UPARSE (version 7.1; http://drive5.com/uparse/).
