# Supplementary material for: Change of soil microbial community under long-term fertilization in a reclaimed sandy agricultural ecosystem
Source: PeerJ. 2019 Feb 27;7:e6497. doi: 10.7717/peerj.6497 (PMC6397634; doi:10.7717/peerj.6497)
Supplement: Supplemental Information 1 [file peerj-07-6497-s001.docx]

**Supplementary methods**

**PCR reaction and Illumina MiSeq sequencing**

The used primers and sequencing platform are listed as the following table 1. PCR was performed using 20-μL reaction mixture containing 5 μL of 2× Taq PCR Master Mix (Sangon Biotech, Shanghai, China), 1 μL of each primer (10 μM), 1 μL of template DNA (10 ng), and sterilized ultrapure water. The PCR conditions were as follows: 94 °C for 4 min, followed by 35 cycles of 94 °C for 30 s, annealing temperature for 30 s, and 72 °C for 30 s, and a final extension at 72 °C for 10 min. The amplicons were separated by 1% agarose gel electrophoresis, purified using SanPrep Column DNA Gel Extraction Kit (Sangon Biotech), and quantified with QuantiFluor™-ST (Promega Corporation, Madison, WI, USA). Purified amplicons were pooled in equimolar and paired-end sequenced (2 × 300) on an Illumina MiSeq platform according to the standard protocols at Majorbio Bio-Pharm Technology Co. Ltd., Shanghai, China (http://www.majorbio.com).

**Processing of sequencing data**

Raw fastq files were demultiplexed, quality-filtered using QIIME (version 1.17) with the following criteria: (i) The 300 bp reads were truncated at any site receiving an average quality score <20 over a 50 bp sliding window, discarding the truncated reads that were shorter than 50bp. (ii) exact barcode matching, 2 nucleotide mismatch in primer matching, reads containing ambiguous characters were removed. (iii) only sequences that overlap longer than 10 bp were assembled according to their overlap sequence. Reads which could not be assembled were discarded.

Operational Units (OTUs) were clustered with 97% similarity cutoff using UPARSE (version 7.1 <http://drive5.com/uparse/>) and chimeric sequences were identified and removed using UCHIME. The taxonomy of each 16S and 18S rRNA gene sequence was analyzed by Silva database (Release128 http://www.arb-silva.de) using confidence threshold of 70% (Amato et al., 2013). Hierarchical clustering analysis was performed using CLUSTER and visualized using TREEVIEW, and other statistical analyses were performed with the IEG pipeline (http://ieg.ou.edu). The average data were calculated for BSCs of each revegetation before analyzing the unique and shared OTUs/genera. The figures were generated with SigmaPlot 11.0 and Excel. Alpha diversity analysis was used to reflect the richness and diversity of microbial communities. In order to investigate the overall differences in community composition among the samples, principle coordinate analysis was performed using unweighted UniFrac distance (Lozupone and Knight, 2005).

**qPCR analysis**

qPCR was performed to determine the absolute 16S and ITS rRNA gene abundance, respectively. We used the primer sets of 27F (5′- AGAGTTTGATCCTGGCTCAG -3′) and 338R (5′- TGCTGCCTCCCGTAGGAGT -3′) for bacteria and ITS1F (5′- CTTGGTCATTTAGAGGAAGTAA -3′) ITS2R (5′-GCTGCGTTCTTCATCGATGC -3′) for fungi to quantify the total microbial biomass. qPCR reactions were conducted in a 20 μL reaction system contained 10 μL of 2 × SYBR mix (with ROX) (DBI Bioscience, Ludwigshafen, Germany), 0.4 μL each of 10 μM forward and reverse primers, 1 μL of total DNA template (1 ng μL^-1^) and 8.2 μL of RNase-free ddH_2_O. The reaction was conducted on a Stratagene Mx3000P real-time PCR system (Stratagene, Agilent Technologies Inc., Santa Clara, CA, USA) using the following program: 50 °C for 2 min and 94 °C for 10 min followed by 45 cycles of 94 °C for 1 min, 55 °C for 1 min and 72 °C for 1 min, and then 72 °C for 2 min. ddH_2_O was used to instead of DNA templates as negative control. The detection signal was collected at 72 °C for 30 s and analyzed. DNA quantitative controls were generated by amplifying the gene from total DNA. The PCR products were purified using the PCR clean up kit (Axygen, USA) and cloned into the pUCm-T vector (Sangon Biotech Shanghai Co., Ltd., Shanghai, China). DNA standards were then made from the plasmid DNA with concentrations ranged from 6.13×10^8^ to 6.13×10^4^ copies of DNA per reaction. The gene copy numbers of the target samples for each reaction were calculated from the standard curves. The melting curve was obtained to confirm that the amplified products were of the appropriate size. For each sample, the qPCRs were replicated six times.

**Table 1.** Information of selected primers and Miseq platform.

| **Sequence type** | **Primer name** | **Primer sequence** | **Amplified sequence length** | **References** | **Sequencing platform** |
| --- | --- | --- | --- | --- | --- |
| Universal primers for bacteria and archaea 16S rRNA | ArBa515F | 5'-GTGCCAGCMGCCGCGGTAA-3' | 291 bp | Scott et al., 2011 | PE250 platform |
|  | Arch806R | 5'-GGACTACVSGGGTATCTAAT-3' |  |  |  |
| Fungi 18S rRNA | SSU0817F | 5'-TTAGCATGGAATAATRRAATAGGA-3' | 379 bp | Rousk et al., 2010 | PE300 platform |
|  | 1196R | 5'-TCTGGACCTGGTGAGTTTCC-3' |  |  |  |

**References**

Amato KR, Yeoman CJ, Kent A, Righini N, Carbonero F, Estrada A, Gaskins HR, Stumpf RM, Yildirim S, Torralba M, Gillis M, Wilson BA, Nelson KE, White BA, Leigh SR (2013) Habitat degradation impacts black howler monkey (Alouatta pigra) gastrointestinal microbiomes. ISME J 7(7): 1344–1353. https://doi.org/10.1038/ismej.2013.16

Bates ST, Berg-Lyons D, Caporaso JG, Walters WA, Knight R, Fierer N (2011) Examining the global distribution of dominant archaeal populations in soil. ISME J 5(5): 908–917. https://doi.org/10.1038/ismej.2010.171

Lozupone C, Knight R (2005) UniFrac: a new phylogenetic method for comparing microbial communities. Applied Environmental Microbiology 71(12): 8228–8235. https://doi.org/10.1128/aem.71.12.8228-8235.2005

[Rousk J](https://www.ncbi.nlm.nih.gov/pubmed/?term=Rousk%20J%5BAuthor%5D&cauthor=true&cauthor_uid=20445636), [Baath E](https://www.ncbi.nlm.nih.gov/pubmed/?term=B%C3%A5%C3%A5th%20E%5BAuthor%5D&cauthor=true&cauthor_uid=20445636), [Brookes PC](https://www.ncbi.nlm.nih.gov/pubmed/?term=Brookes%20PC%5BAuthor%5D&cauthor=true&cauthor_uid=20445636), [Lauber CL](https://www.ncbi.nlm.nih.gov/pubmed/?term=Lauber%20CL%5BAuthor%5D&cauthor=true&cauthor_uid=20445636), [Lozupone C](https://www.ncbi.nlm.nih.gov/pubmed/?term=Lozupone%20C%5BAuthor%5D&cauthor=true&cauthor_uid=20445636), [Caporaso JG](https://www.ncbi.nlm.nih.gov/pubmed/?term=Caporaso%20JG%5BAuthor%5D&cauthor=true&cauthor_uid=20445636), [Knight R](https://www.ncbi.nlm.nih.gov/pubmed/?term=Knight%20R%5BAuthor%5D&cauthor=true&cauthor_uid=20445636), [Fierer N](https://www.ncbi.nlm.nih.gov/pubmed/?term=Fierer%20N%5BAuthor%5D&cauthor=true&cauthor_uid=20445636) (2010) Soil bacterial and fungal communities across a pH gradient in an arable soil. ISME J. 4(10): 1340-1351. https://doi.org/10.1038/ismej.2010.58
