# Supplementary figures and images for: Change of soil microbial community under long-term fertilization in a reclaimed sandy agricultural ecosystem
Source: PeerJ. 2019 Feb 27;7:e6497. doi: 10.7717/peerj.6497 (PMC6397634; doi:10.7717/peerj.6497)

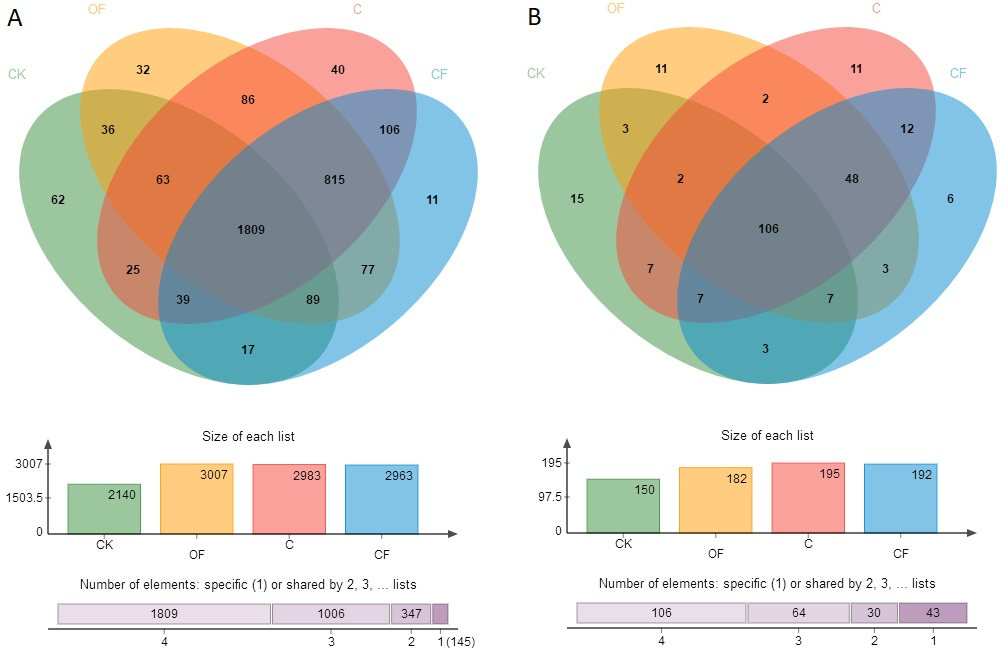

Supplement: Figure S1 [file peerj-07-6497-s002.png]
