# Supplementary material for: Change of soil microbial community under long-term fertilization in a reclaimed sandy agricultural ecosystem
Source: PeerJ. 2019 Feb 27;7:e6497. doi: 10.7717/peerj.6497 (PMC6397634; doi:10.7717/peerj.6497)
Supplement: Table S1 — Details of crop agronomic characteristics under different fertilization treatments. [file peerj-07-6497-s003.docx]

**Table. S1** Details of crop agronomic characteristics under different fertilization treatments.

|  |  | C | CF | OF |
| --- | --- | --- | --- | --- |
| Above ground biomass (kg/m^2^) |  | 1.66±0.06 ^c^ | 2.47±0.11 ^a^ | 2.13±0.13 ^b^ |
| Underground biomass (kg/m^2^) |  | 0.16±0.03 ^b^ | 0.21±0.02 ^a^ | 0.19±0.03 ^a^ |
| Seed yield (g/m^2^) |  | 307.97±3.73 ^b^ | 523.39±12.17 ^a^ | 492.19±0.48 ^a^ |
| Leaf area index |  | 2.12±0.11 ^b^ | 3.39±0.13 ^a^ | 3.27±0.1 ^a^ |
| Stem length (cm) |  | 68.37±2.85 ^b^ | 79.5±1.80^a^ | 78.7±4.01 ^a^ |
| Thousand seeds weight (g) |  | 17.43±1.21 ^b^ | 21.23±1.04 ^a^ | 20.87±0.12 ^a^ |

The significant differences in each parameter (mean±SE) among different treatments were determined by one-way ANOVA (LSD test). Different letters indicate significant difference at P<0.05.
